# xTMS: A Pulse Generator for Exploring Transcranial Magnetic Stimulation Therapies

**DOI:** 10.1109/APEC43580.2023.10131554

**Published:** 2023-05-31

**Authors:** Kawsar Ali, Karen Wendt, Majid Memarian Sorkhabi, Moaad Benjaber, Timothy Denison, Daniel J. Rogers

**Affiliations:** 1 Department o fEngineering Science, University of Oxford, UK; 2 MRC Brain Network Dynamics Unit, Nuffield Department of Clinical Neurosciences, University of Oxford, UK

**Keywords:** CCPS, CHB, MMC, MPC, TMS, transcranial magnetic stimulation, pulse power

## Abstract

A cascaded H-bridge based pulse generator for transcranial magnetic stimulation is introduced. The system demonstrates complete flexibility for producing different shape, duration, direction, and rate of repetition of stimulus pulses within its electrical limits, and can emulate all commercial and research systems available to-date in this application space. An offline model predictive control algorithm, used to generate pulses and sequences, shows superior performance compared to conventional carrier-based pulse width modulation. A fully functioning laboratory prototype delivers up to 1.5 kV, 6 kA pulses, and is ready to be used as a research tool for the exploration of transcranial magnetic stimulation therapies by leveraging the many degrees-of-freedom offered by the design.

## I Introduction

NEW tools and methods for non-surgical brain stimulation are needed to better probe and resolve human brain activity dynamics in health and disease.Transcranial magnetic stimulation(TMS), an FDA-cleared therapy for treatment-resistant depression, is one such method that uses electro-magnetic induction to stimulate the neurons in localised areas of the brain [1]. TMS offers distinct advantages compared to other non-surgical brain stimulation techniques such as electroconvulsive therapy(ECT) [2] and transcranial direct current stimulation (tDCS) [3]. However, generating bespoke magnetic stimuli is still a considerable challenge for TMS, which has in turn impeded the systematic investigations of the effects of TMS stimuli on neuronal dynamics and brain behaviour [4].

Traditionally, TMS is achieved by a simple LC resonator [5], [6], which has limitations in shape, duration, direction, pattern, and rate of repetition of stimulus pulses. H-bridge circuits made of high-voltage, high-current transistors combined with Pulse Width Modulation (PWM) offer more flexibility in almost all of these aspects [7]–[11]. Although such circuits have gained momentum recently in TMS applications, they are still in the research stage. This is partly because they produce a PWM approximated sinusoidal pulse as opposed to a smoother sinusoidal pulse generated from an LC resonator, and the effects of the high-frequency harmonics present in the PWM pulse on the neuron are still under study [12]. Modular Multilevel Converters (MMC) can approximate the sinusoidal pulse (or any other pulse-shape) more closely than a single H-bridge [13]–[16]. Additionally, MMCs offer scalability and flexibility for design and operation of high power systems like TMS.

The simplicity offered by the MMCs is, however, often complicated in their reported applications in TMS. For example, an obvious compromise in [14] is the paralleling of the dc-link capacitors of two or more H-bridges to generate a lower intensity output pulse, which might otherwise be achieved by improved modulation. To connect the capacitors in parallel, their voltages must be balanced using complex control [15]. The charging scheme implemented in [16] causes unwanted inrush current through the stimulus coil because of unbalanced capacitor voltages. Similarly, the pre-charging circuit presented in [13] charges the dc-link capacitors in parallel, which can cause circulating current related issues if the capacitor voltages are not equal, e.g., after delivering a stimulus pulse. A Cascaded H-bridge (CHB) inverter with isolated dc source for each H-bridge module can eliminate all of these issues [11], [17].

Usually, the H-bridges are modulated using carrier-based PWM, where the target output reference signal (normalised) is compared with a triangular carrier signal to generate the gate pulses for the transistors in the H-bridge. A high-fidelity output is achieved when the carrier frequency is much higher (≥20×) than the reference frequency and the dc-link voltage variation is negligible. However, for high-power pulsed-load applications like TMS, where the peak-to-average power ratio is very high, the dc-link voltage is allowed to drop by up to 50% of the initial voltage to reduce the required dc-link capacitance. Also, the practical limit on the maximum switching frequency of commercial high power IGBTs is limited (∼20 kHz) and close to the reference frequency of 2-5 kHz for TMS pulses. The combined effect of these two limitations is a significant deviation of the TMS output pulse from its reference (see section III) when conventional carrier-based PWM method is used. Efforts have been made to compensate for this deviation by modifying the reference signal itself [14], although this is not a systematic approach and may give variable results depending on pulse shape. An offline proportional-integral (PI) control is proposed in [18] to minimize the TMS pulse-current distortions in CHB-based TMS systems. However, it compensates only for the distortions caused by parasitic power losses in the coil and IGBTs, and not for the deviations caused by dc-link voltage drop and limited switching frequency, as discussed above. Additionally, complex programming of the module states is required for actively snubbing the ringing in the coil at the end of output pulses [16].

Model Predictive Control (MPC) based PWM alleviates these problems by treating the PWM generation as an optimisation problem over a time horizon. By taking the dc-link voltage and other circuit states as feedback at each time step up to the horizon, MPC determines the next switching state to generate the target output pulse. Naturally, Online MPC is challenging because it requires far more computing resource than carrier-based PWM. However, pulsed power applications like TMS are characterised by short, discrete, and non-periodic outputs, and thus, do not need online MPC. Both the small-signal and large-signal dynamics of the system are well-defined and can be included in an offline MPC calculation.

In this paper, a next-generation transcranial magnetic stimulation (xTMS) research tool is introduced that has unmatched stimulus control capabilities. It can be used in single-pulse, paired-pulse or repetitive TMS (rTMS) mode without any circuit modification. The system is fully modular, with each H-bridge module having its own isolated dc-dc converter for charging its dc-link capacitor, thereby eliminating the issues with unbalanced capacitor voltages for the MMC topology. Three modules are cascaded to generate a seven level PWM-approximated stimulus pulse, showing a good cost-benefit trade-off [17]. The system is controlled by an offline (open-loop) MPC algorithm developed in-house. Taking the system specifications, it generates and stores a library of output pulses in advance of use, thereby eliminating the computational challenge of performing MPC in real-time while retaining its advantages.

## II System Design and Operating Principle

**Fig. 1:**
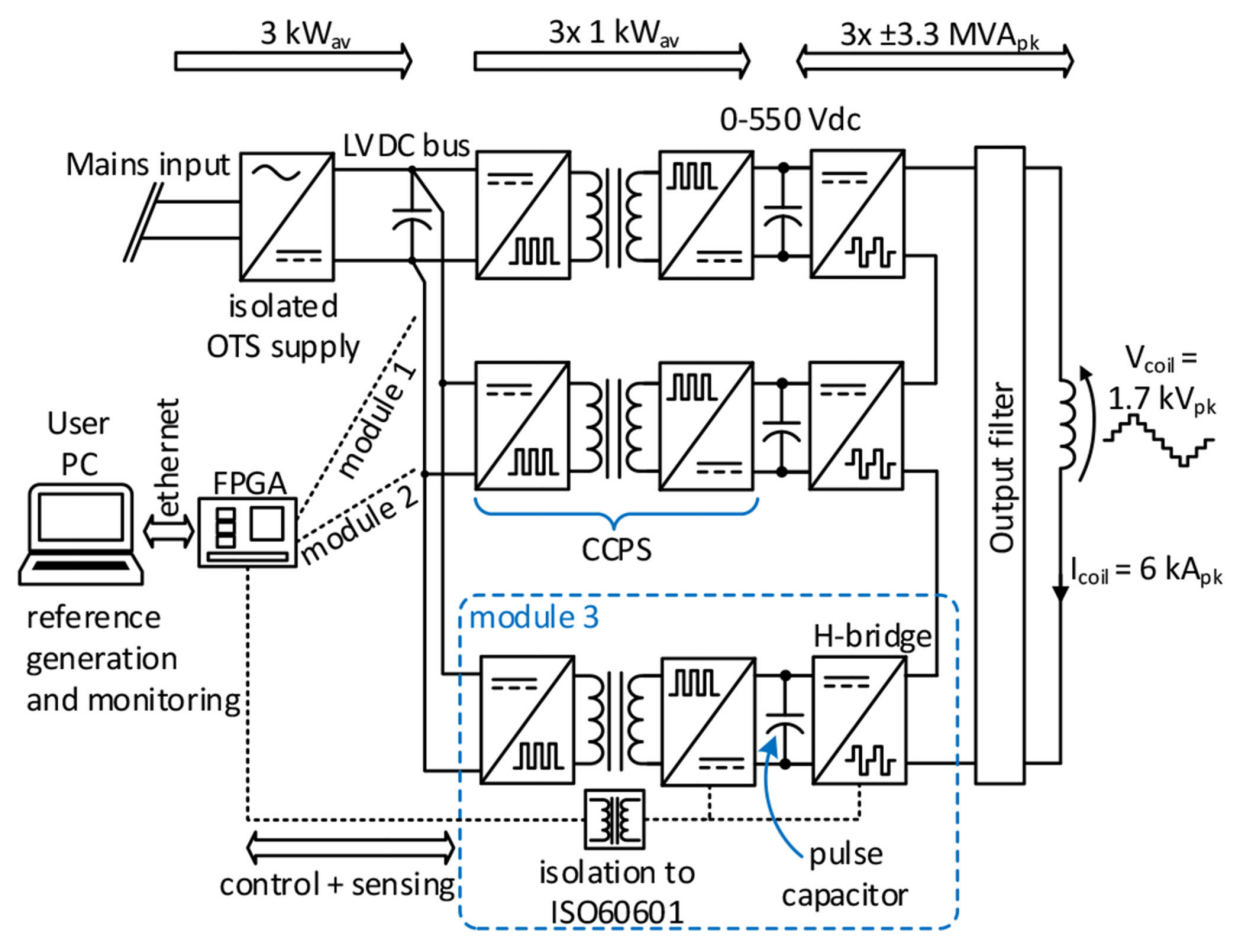
The xTMS system electrical block diagram.

**Fig. 2:**
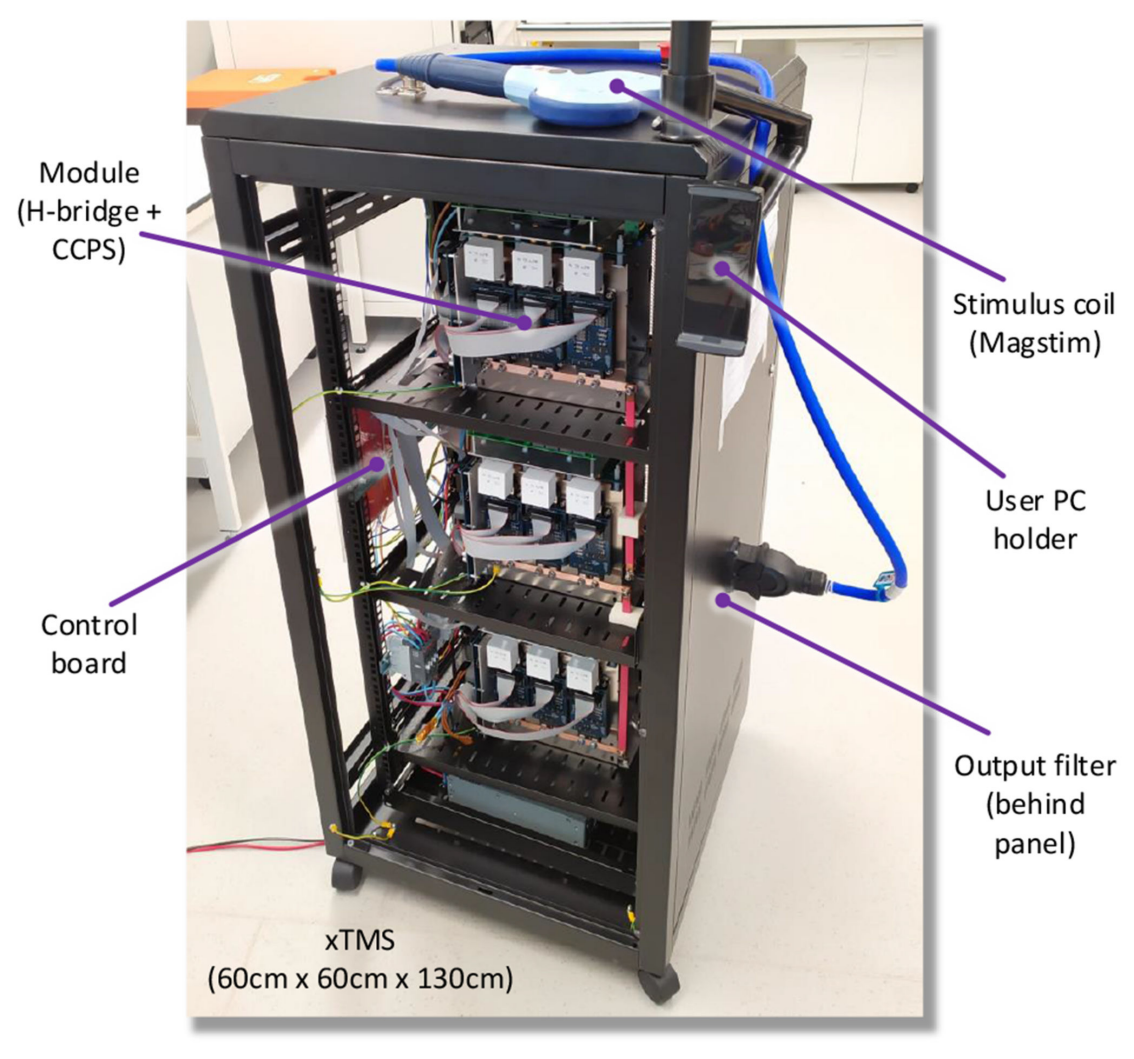
Laboratory prototype of the xTMS system.

The functional block diagram of the xTMS system is shown in Fig. 1. The design converts the mains power to high-power magnetic pulses in three distinct stages. The first stage is the rectification of the mains ac voltage to create a low-voltage dc (LVDC) bus by an off-the-shelf ac-dc power supply. In the second stage, an isolated dc-dc converter charges the energy storinghigh-voltage dc-link capacitors up to 550 V. In the third stage, a 7-level CHB inverter creates the stimulus from the dc-link energy with a non-resonant, high-frequency switching design. The laboratory prototype of the system is shown in Fig. 2.

### A Isolated DC-DC converter

This stage provides medical-grade galvanic isolation (ISO60601) and transforms the LVDC input into a 0-550 V dc variable output. As the load seen by this converter is purely capacitive, this converter stage is also known as the capacitor charging power supply (CCPS). A capacitive load is characterised by a widely varying output voltage from 0 V at the beginning of charging to 550 V at the end. As a result, conventional converters (e.g., Flyback converter, Full-Bridge PWM converter, Dual Active Bridge converter etc.) cannot achieve soft-switching during the whole charging period without a complex control scheme and/or auxiliary circuits, resulting in poor charging efficiency [19]. A resonant converter has been used here for CCPS design as it offers complete soft-switching behaviour with simpler control and lower component count.

### B Cascaded H-bridge(CHB)Inverter

**Fig. 3:**
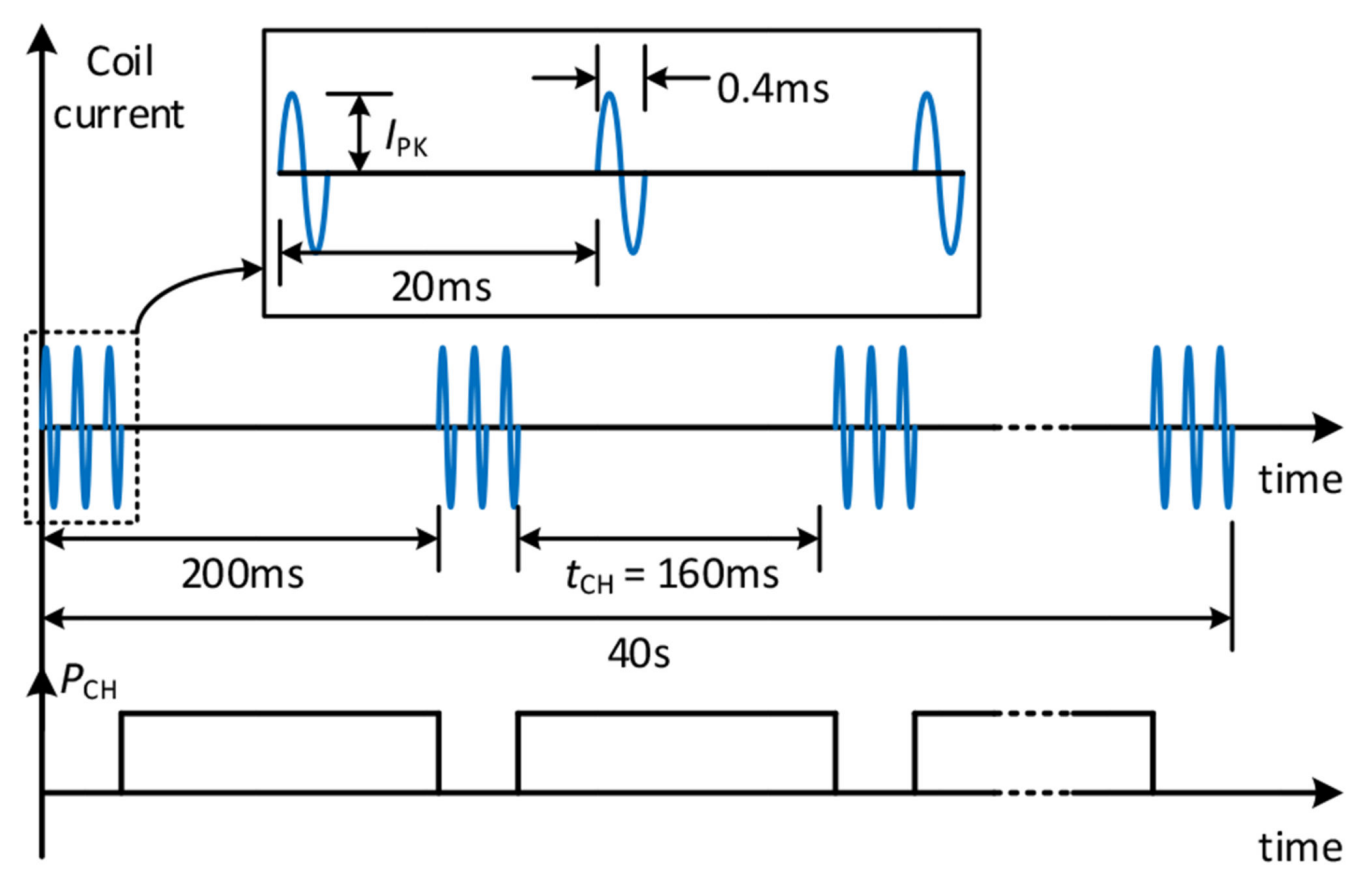
The biphasic continuous theta burst (cTBS) protocol. Each triplet isconsidered a single pulse during which the capacitor charging power suppliesare off.

In this stage, three H-bridge modules in cascade generate a 7-level PWM-approximation of the reference output pulse. Three parallel IGBT half-bridge modules each rated at 1200V/1800A (peak) are used in each leg of each H-bridge. Since the TMS pulses are very short (200-500 *μ*s) and the effective duty ratio of the IGBTs in TMS application is also very small (<1%, see Fig.3), each IGBT can carry 2 kA pulse current (which is 10% higher than the datasheet-specified repetitive peak collector current) without violating its transient thermal limit [20]. Thus, the system can deliver output pulses of up to 3 kV and 6 kA peak amplitudes. To ensure equal current sharing, the three IGBT modules in parallel, are driven by one gate driver connected to three boosters local to each of the three IGBT modules.

Each H-bridge also has its own dc-link capacitor. The MPC modulation approach allows higher depth of discharge of the capacitor voltages without a reduction in output waveform quality. This reduces the capacitance requirement and the dc-link can be implemented using film capacitors (instead of electrolytic capacitors) that have an efficiency and lifetime suited for pulse applications.

### C Output Filter

Operation of the CHB produces sharp-edged rectangular voltage pulses that have significant high frequency content. This should, ideally, be removed by suitable filtering in order to produce the desired “smooth” TMS stimulus waveform. In a TMS application, there are effectively two cascaded low-pass filters naturally present in the system. Firstly, the inductance of the stimulation coil provides filtering through integration of the applied PWM voltage leading to the resulting coil current. Secondly, the neurons respond to the voltage induced by the varying magnetic field created by the coil current and the neural activation response is modelled as low-pass [21]. This is hypothesised to further reduce the effect of high frequency content in the magnetic field produced by the coil current [11].

Additionally, the parasitic capacitances from the stimulus coil and the IGBTs resonate with the coil inductance and create high-frequency ringing in the switching edges of the applied coil voltage. To mitigate potential electromagnetic interference (EMI) issues on other clinical equipment as well as unmodelled neuronal response (which is subject to further research [12]), a filter connected before the stimulus coil is used for attenuating the high frequency content of the output voltage.

### D Electrical Limit

Among various repetitive TMS (rTMS) protocols, the continuous theta burst (cTBS) protocol [22] requires the highest average power from the system. Therefore, the cTBS protocol is chosen to size the xTMS system. In this protocol, as shown in Fig. 3, bursts of 50 Hz triplets are repeated at 5 Hz for 40 seconds continuously.

An average power limit must be satisfied: from a 10 A, 240 V wall socket a maximum of 2400 W average power can be drawn. In order to use the full 2400 W power to charge the capacitors during the inter-burst charging period of cTBS, the dc-link capacitors are charged in constant power (CP) mode (as opposed to constant current (CC) mode) during this inter-burst interval *t*_CH_. Thus, the energy that can be delivered to the capacitors in one cTBS burst is

1 $$E_{TBS}=P_{CP}t_{\mathrm{CH}}$$

where *P*_CP_ = 2400 W is the power limit. For *N*_M_ number of modules in the system each with *C*_M_ dc-link capacitance, the following holds,

2 $$C_{M}({V_{H}}^{2}-V{{}_{L}}^{2})/2=E_{\mathrm{TBS}}/N_{M}$$

where *V*_H_ and *V*_L_ are the capacitor voltages at the beginning and at the end of one burst, respectively. With *P*_CP_, *t*_CH_, *V*_H_, and *N*_M_ given, a combination of *C*_M_ and *V*_L_ can be calculated for the system design. One such feasible design (based on stimulus coil voltage rating, dc-link capacitor availability, and IGBT voltage rating) is shown in Table I.

If there are *N*_P_ pulses in one burst of cTBS, the achievable peak coil current *I*_PK_ for cTBS protocol can be calculated from the following, where *R*_C_ is the coil resistance.

3 $$E_{\mathrm{TBS}}/N_{P}={I_{\mathrm{PK}}}^{2}R_{C}t_{\mathrm{PUL}}/2$$

4 $$I_{PK}=\sqrt{2P_{\mathrm{CP}}t_{\mathrm{CH}}/(N_{P}R_{C}t_{\mathrm{PUL}})}$$

Below *V*_L_ the capacitors should be charged in the constant current (CC) mode so as to not violate the current ratings of the components (transistors, diodes, resonant capacitors, and transformer) of the CCPS. The charging current in CC modeis given by

5 $$I_{CC}=P_{\mathrm{CP}}/(N_{M}V_{L}).$$

Therefore, the CCPS must be rated at a power of at least

6 $$P_{CCPS}=V_{H}I_{CC}.$$

Finally, the capacitor charging time at the start-up of the system can be calculated as

7 $$t_{\mathrm{START}}=C_{M}(V_{L}-V_{0})/I_{CC}+C_{M}({V_{H}}^{2}-{V_{L}}^{2})/(2P_{\mathrm{CP}})$$

where *V*_0_ is the initial capacitor voltage before the start-up.

## III Offline Model Predictive Control(MPC)

Model predictive control is a framework for choosing the optimal way to operate a system to achieve an objective, subject to a set of constraints. For the xTMS system these are listed below.

- Objective: Cause the desired output current to flow in the stimulus coil (even if there is a complex filter between the IGBTs and the coil) when the dc-link capacitor voltage is continually varying as energy is transferred.
- Constraints: Do not switch the IGBTs too often, do not allow the dc-link capacitor voltages to become too unbalanced.
- Result: The best waveform quality for a given number of IGBT switching events.

In the context of power electronics, MPC is typically discussed as an online closed-loop feedback system for control of continuous output. In such systems, measurements of states of the system are used to correct for the model inaccuracies that would otherwise accumulate over time. In contrast, offline MPC is an open-loop approach. By ensuring the availability of an accurate state-space model of the system, the proposed MPC algorithm is implemented offline to generate the gate signals for the IGBTs to produce the desired pulse shape. Since the relation between the dc-link voltage and the coil current is linear, the pulse intensity is easily varied by controlling the dc-link voltage via the CCPS.

**Fig. 4:**
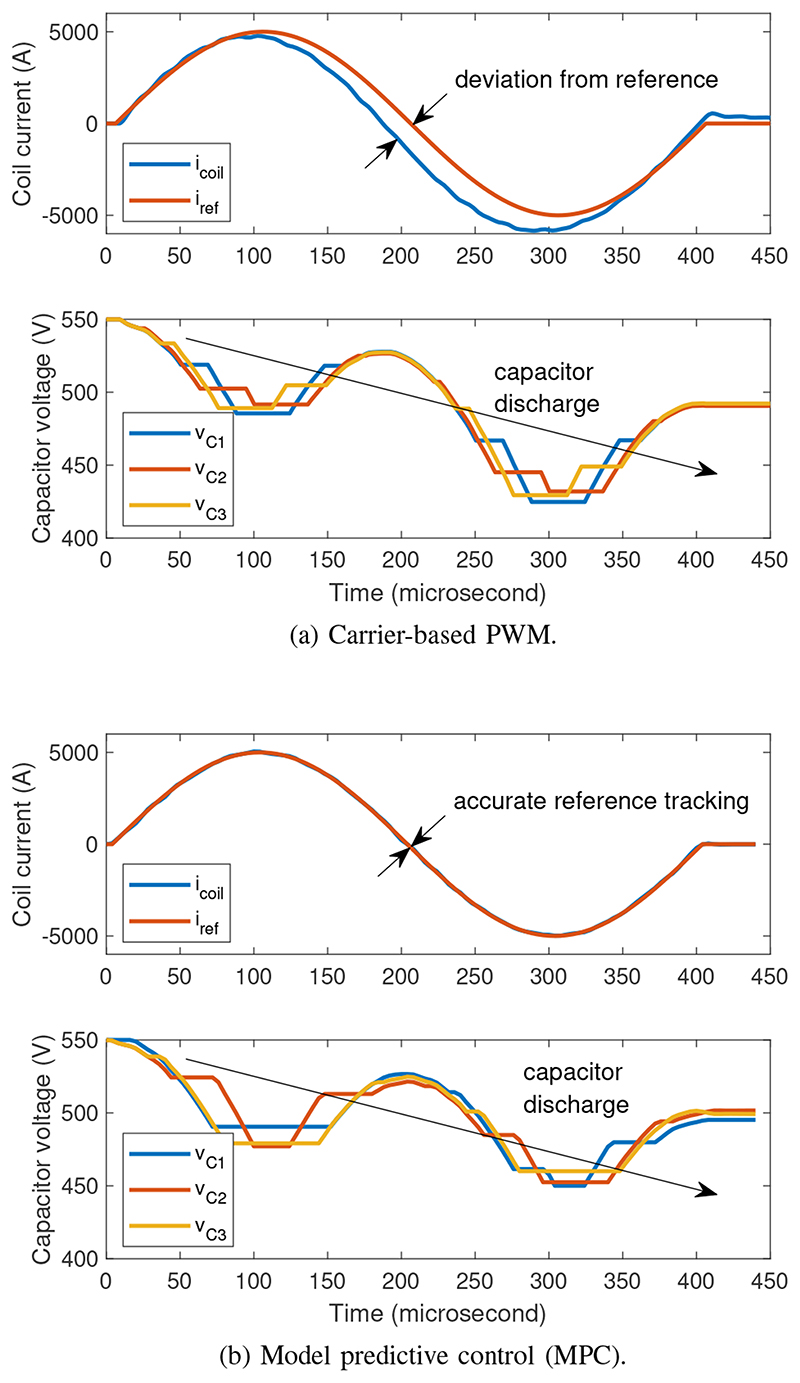
Comparison of pulse quality between carrier-based PWM and model predictive control (MPC) in simulation. Even with decreasing capacitor voltages the MPC generated pulse closely follows the reference.

A comparison between the conventional carrier-based PWM and the proposed MPC in terms of the quality of the generated output pulse is shown in Fig. 4. As the dc-link capacitor voltages decrease, the output pulse generated by carrier-based PWM deviates from the reference, whereas the MPC generated pulse follows the reference throughout the whole pulse duration.

The control system for the xTMS system is developed on a FPGA System-on-Chip platform.

## IV Experimental Results and Discussions

The xTMS system was tested with a module dc-link voltage of up to 550 V and a peak coil current of up to 6 kA. It can deliver pulses of width varying from zero to several milliseconds, limited by the energy available from the dc-link capacitors and the maximum rate-of-change of coil current supported by the design. The maximum energy transferred to the figure-eight coil (Magstim Ltd.) was 288 Joules. The coil voltage and current were measured using a a high-voltage differential probe (TA044, PICO TECHNOLOGY) and a Rogowski current probe (I6000S FLEX-24, FLUKE), respectively. All measurements were performed on a digital oscilloscope with a sampling rate of 250 MSa/s. No bandwidth restrictions or filters have been applied.

**Fig. 5:**
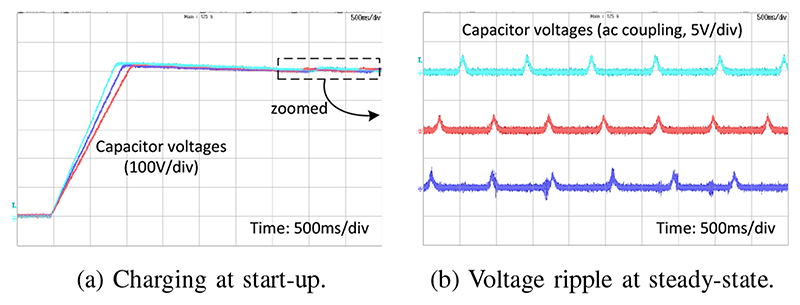
Charging and voltage control of the dc-link capacitors at the start-up and the steady-state of the system.

Fig. 5 shows the charging of the dc-link capacitors at system start-up. It takes about one second to charge the 2.62 mF capacitor from 0 to 500 V with a constant charging current of 1.5 A. After reaching steady-state, the voltage oscillates about its reference value with a ±1% ripple where the CCPS operates in burst-mode voltage control. The burst mode compensates for the continuous discharge through the safety discharge resistor connected across the dc-link capacitor. The very low voltage ripple ensures that the offline MPC has an accurate model of the initial state of the system to deliver the desired pulse with minimum deviation from the reference.

**Fig. 6:**
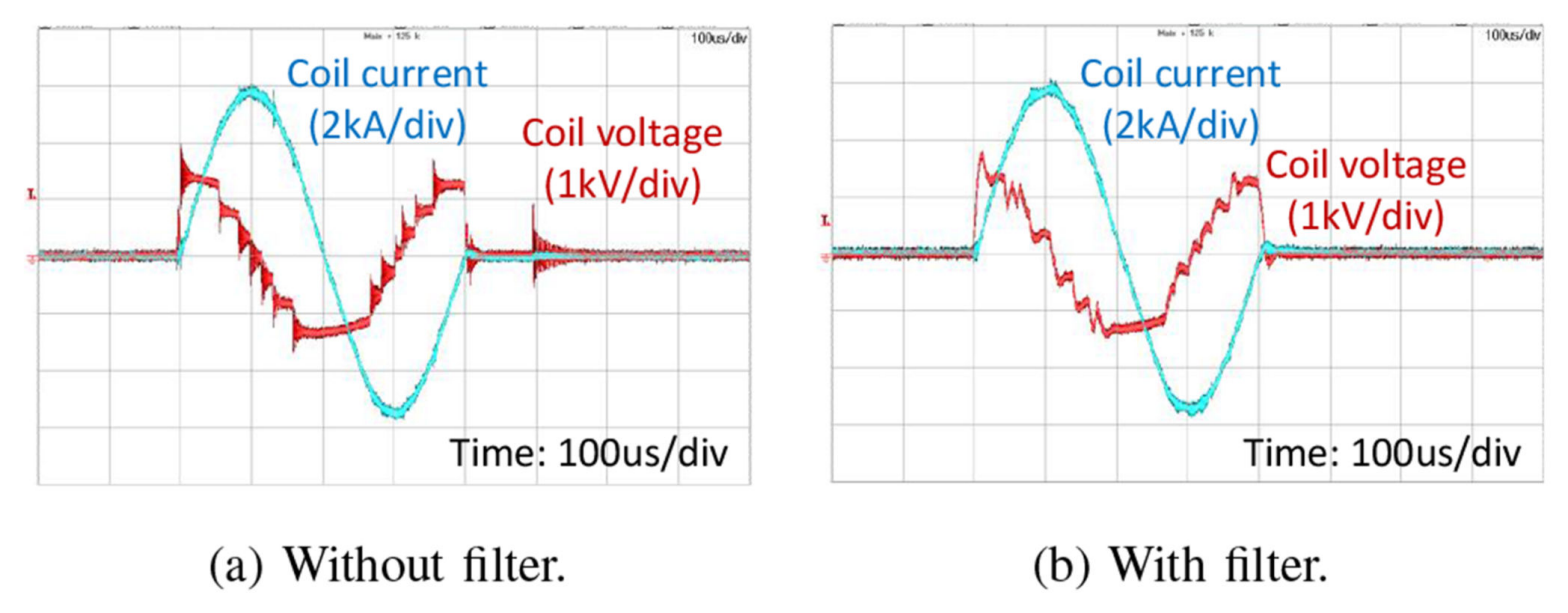
A biphasic pulse with and without the output filter showing the efficacy of the output filter to eliminate high frequency noise from the coil voltage waveform.

The significance of the output filter is demonstrated in Fig. 6. A 60 kHz cut-off frequency attenuates the high frequency content of the coil voltage waveform.

**Fig. 7:**
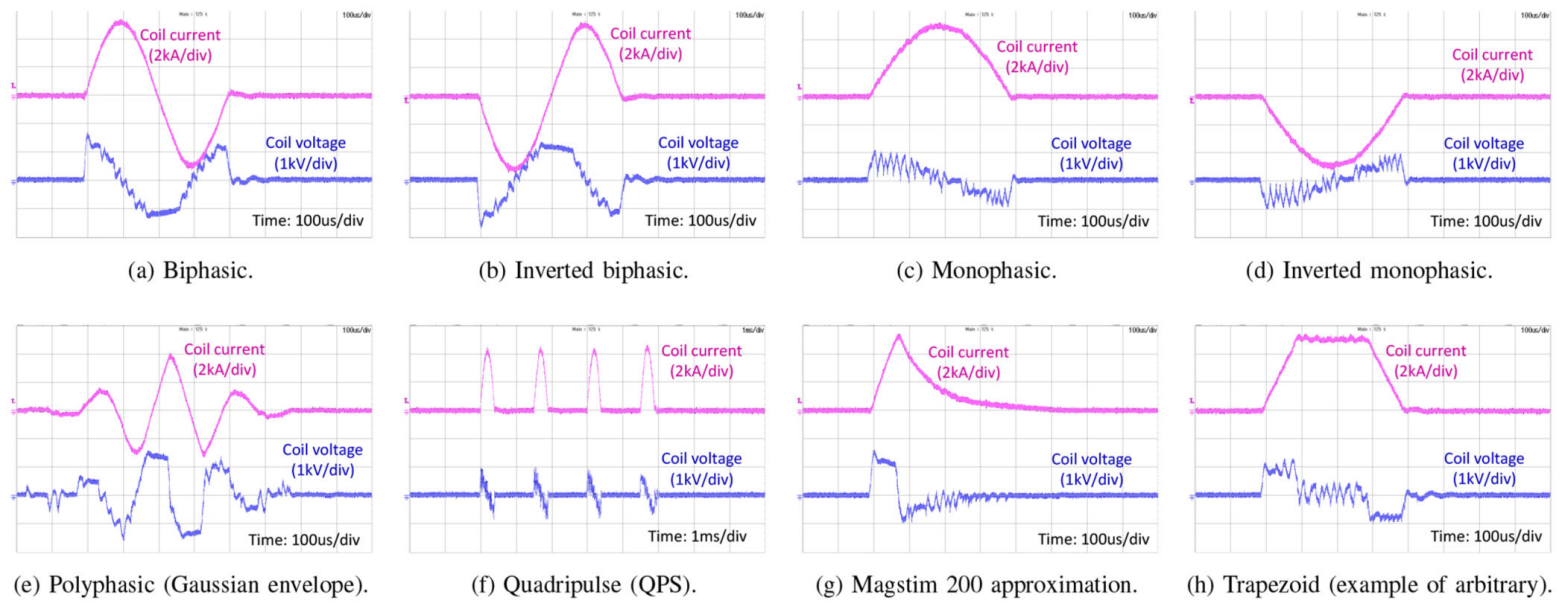
A sample set of conventional and arbitrary pulse shapes demonstrating the capability of xTMS.

Fig. 7 presents a sample set of conventional and arbitrary shapes of stimulus waveforms that can be generated by the xTMS system. These cover all the pulses generated by commercial systems, as well as selected pulses shown in research systems. The system can generate any pulse shape as long as the electrical limits (capacitor energy, peak current, peak voltage) are satisfied. In contrast to commercial TMS equipment (e.g., BiStim^2^ and Rapid^2^ from MAGSTIM), for which conventionally the output of several TMS devices must be combined, a single xTMS machine can generate rapid repetitive protocols like quadripulse stimulation (QPS) [23] as shown in Fig. 7.

**Fig. 8:**
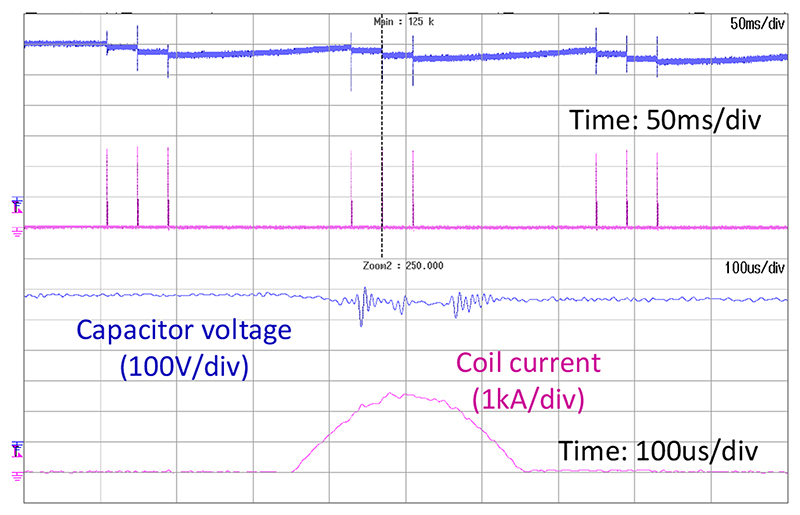
Monophasic continuous theta burst (cTBS) protocol delivered by xTMS.

Fig. 8 shows an example of the xTMS system’s capability in generating repetitive protocols such as cTBS with monophasic pulses. It also shows how the dc-link capacitor is charged back up to the reference voltage in between the bursts of triplets.

**Fig. 9:**
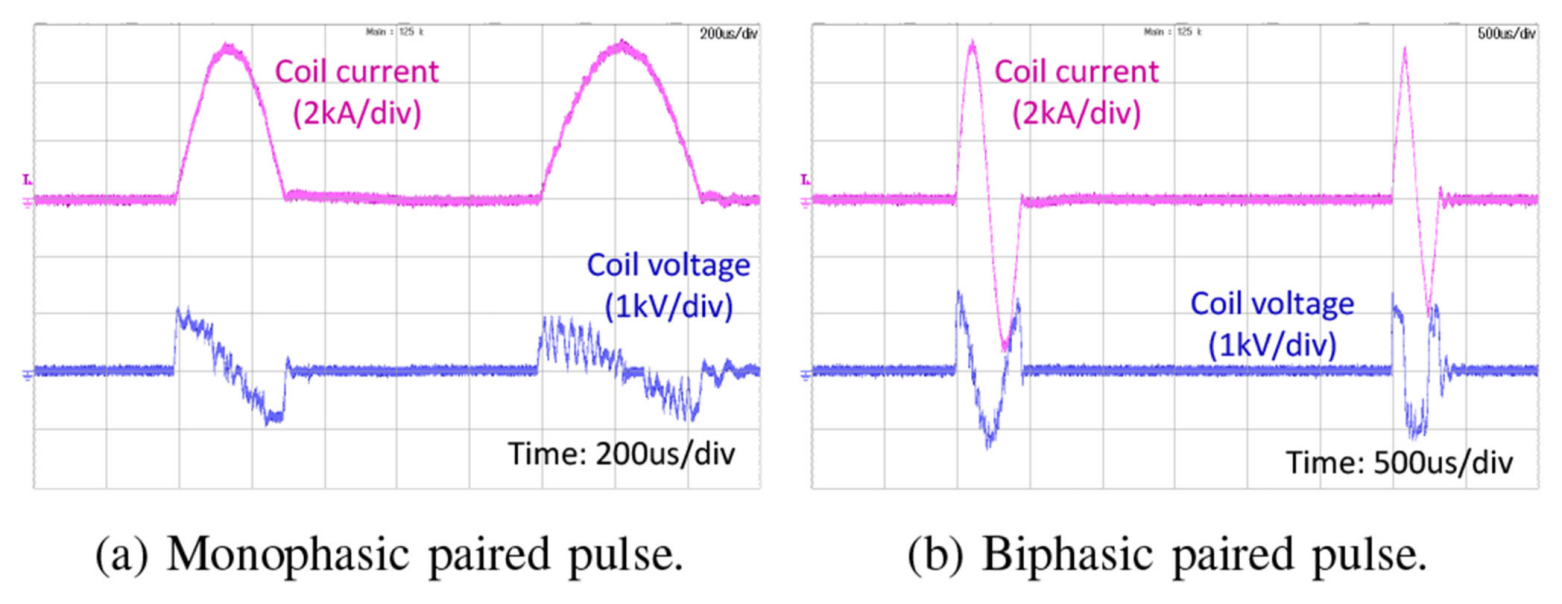
Examples of paired pulse delivered by xTMS.

Fig. 9 shows examples of paired pulses with (a) monophasic and (b) biphasic pulses. The size and shape of the first and second pulse can be altered independently allowing for flexibility within the protocol.

## V Conclusions

A cascaded H-bridge configuration, where each module has its own charging circuit, is a cost-effective route to obtain a scalable and flexible TMS system. The extremely high peak-to-average power ratio of TMS therapy poses challenges in producing high-fidelity outputs when using conventional carrier-based PWM. An offline MPC approach is shown to overcome this challenge without adding any additional complexity to the system. Within its electrical limit, the xTMS can generate any pulse-shape or pulse-sequence enabling a systematic exploration of TMS therapies.

## Figures and Tables

**Table 1:** System Sizing Based On Electrical Limit

Parameter	Design value
Given:
Maximum available power (*P*_CP_)	2400 W
Inter-burst charging time for cTBS (*t*_CH_)	160 ms
No. of modules (*N*_M_)	3
No. of pulses in one cTBS burst (*N*_P_)	3
One pulse duration (*t*_PUL_)	400 *μ*s
Coil resistance (*R*_C_)	37 mΩ
Calculated:
Capacitor voltage at the start of one cTBS burst (*V*_H_, assumed)	550 V
Energy delivered in one cTBS burst (*E*_TBS_)	384 J
Capacitor voltage at the end one cTBS burst (*V*_L_, assumed)	450 V
Module capacitance needed (*C*_M_)	2.6mF
Charging current in CC charging mode (*I*_CC_)	1.77 A
CCPS power rating (*P*_CCPS_)	977 W
Achievable coil peak current for cTBS protocol (*I*_PK_)	4159 A
Charging time at start-up of the system (*t*_START_)	0.72 s
